# Sex differences in 10-year ischemic cardiovascular disease risk prediction in Chinese patients with prediabetes and type 2 diabetes

**DOI:** 10.1186/s12872-019-1232-y

**Published:** 2019-12-18

**Authors:** Lihong Yang, Anne F. Fish, Yuanyuan Zhu, Xiaodan Yuan, Jianing Li, Xiaoyun Wang, Li Yuan, Zhumin Jia, Chao Liu, Yunchen Xu, Qingqing Lou

**Affiliations:** 1 Department of Health Education, Affiliated Hospital of Integrated Traditional Chinese and Western Medicine, Nanjing University of Chinese Medicine, #100 Hongshan Road, Qi xia District, Nanjing, Jiangsu Province, 210028 China; 2 Jiangsu Province Academy of Traditional Chinese Medicine, 100 Shizi Street, Hongshan Rd, Qi Xia District, Nanjing, Jiangsu Province, China; 3 Eastern Branch of Suzhou Municipal Hospital, 16 Baita West Rd, Gu Su District, Suzhou, Jiangsu Province, China; 4 College of Nursing (ISP Program), University of Missouri-St. Louis, One University Blvd, M/C 529, St. Louis, MO USA; 5 Nursing College, Nanjing University of Chinese Medicine, 138 Xianlin Rd, Qi Xia District, Nanjing, Jiangsu Province, China; 6 Department of Endocrinology, Shanxi Provincial People’s Hospital, 29 Twin Towers Temple Street, Taiyuan, Shanxi Province China; 7 Department of Endocrinology, West China Hospital, West China School of Medicine, Sichuan University, 37 Guoxue Lane, Wu Hou District, Chengdu, Sichuan Province China; 8 Department of Endocrinology, Affiliated Hospital of Henan University of Science and Technology, 24 Jinghua Rd, Luoyang, Henan Province China; 9 Hainan People’s Hospital-Longhua, #8 Longhua Rd, Longhua District Hainan, Haikou, 570102 China

**Keywords:** Cardiovascular disease, Risk assessment, Duration of diabetes, Prediabetes, Case control studies

## Abstract

**Background:**

Cardiovascular disease has become a serious public health problem in recent years in China. The aim of the study was to examine sex differences in cardiovascular risk factors and 10-year ischemic cardiovascular disease (ICVD) risk in Chinese patients with prediabetes (PreDM) and type 2 diabetes mellitus (T2DM).

**Methods:**

This was a multi-site retrospective case-control study conducted from April–November 2016 using an electronic medical record database, involving 217 PreDM and 900 T2DM patients admitted to endocrinology units in four hospitals in China. CVD risk was estimated using the Chinese 10-year ICVD risk model. The differences in 10-year absolute ICVD risk according to PreDM, T2DM < 1 year, T2DM 1–5 years or T2DM ≥5 years and sex were analyzed using ANOVA.

**Results:**

When compared to PreDM females, males with PreDM had significantly higher 10-year ICVD risk In contrast, the opposite pattern of 10-year ICVD risk was observed in T2DM; males had significantly lower 10-year ICVD risk. Moreover, compared to T2DM females, males with T2DM had a lower proportion s with moderate or greater ICVD risk (*p* < 0.001). When compared to PreDM males, males with T2DM < 1 year, and with T2DM 1–5 years had no difference in 10-year ICVD risk, but had higher ICVD risk with T2DM ≥5 years (*p* < 0.05). Compared to PreDM females, females with T2DM in all subgroups had higher ICVD risk (p < 0.05). Among those with T2DM, hypertension rates of awareness, treatment and control were 78.60%, 65.38% and 31.10%, respectively; hyperlipidemia rates of awareness, treatment and control were lower (29.15%, 8.30% and 3.47%, respectively). Females with T2DM had higher prevalence, awareness and treatment of hypertension and hyperlipidemia than males with T2DM (*p* < 0.001).

**Conclusions:**

There is a greater need for cardiovascular risk reduction programs for females with T2DM at diagnosis. Given the low numbers for awareness, treatment and control of hypertension and hyperlipidemia in both males and females, significant resources focused on them must be expended, specifically improving regular assessment of blood pressure and blood lipids. Strengthening the management of chronic diseases through adherence to evidence-based guidelines to enhance clinical treatment may reduce 10-year ICVD in patients with T2DM in China.

## Background

In 2017, the International Diabetes Federation reported that China had the highest number of adults (20–79 years) with diabetes mellitus. Its Diabetes Society refers to China as the global epicenter of the diabetes epidemic. About 114.4 million Chinese adults have diabetes, and its prevalence is expected to rise to 119.8 million by 2045 [1]. Overall, the prevalence of type 2 diabetes mellitus (T2DM) is similar in women and men in China, the prevalence among men was 12.1% and among women was 11.0% [2]. Prediabetes (PreDM) has dramatically increased in China, rising from 15.5% in 2007 to 50.1% in 2010 [2, 3]. According to the 20-year China Da Qing Diabetes Prevention Study [4], 93% of persons with PreDM will progress to overt T2DM in 20 years, imposing a large health and economic burden on China. In addition, PreDM is also an important risk factor for the development of cardiovascular disease [5].

Recently, ischemic cardiovascular disease (ICVD) is increasing in China, although it appears to have fallen over the past 2 decades in the US and Europe [6]. In 2013, the age-standardized mortality rate for ischemic heart disease increased by 2.6% and for stroke increased by 28.8% [7]. About 2 out of every 5 deaths is from cardiovascular disease, the leading cause of death in China [8]. Some evidence suggests that T2DM confers a stronger excess risk of cardiovascular diseases in women than in men [9, 10], yet a National Health and Nutrition Examination Survey (NHANES) [11] in the US showed that women in the midlife years have historically been at a lower risk for overall vascular events than similarly aged men. Therefore, examining sex differences in ICVD risk and preventing ICVD in China in those already diagnosed with T2DM are important goals in diabetes care.

A Chinese task group of the national Fifteen Project developed sex-specific optimal 10-year risk prediction models in the China MUCA study cohort II [12]. Distinct from models developed in Europe, the Chinese 10-year ICVD risk model estimates total ICVD risk (both coronary heart disease and stroke). It is appropriate for Chinese racial and ethnic groups and has been incorporated into current Chinese guidelines for cardiovascular disease prevention. Although several studies have used the 10-year ICVD risk in Chinese adults, its prediction of sex differences in those with PreDM and T2DM has not been reported [13–15]. Hence, in the current study, we examined CVD risk factors and used an established ICVD tool to examine the 10-year ICVD risk in male and female Chinese adults with PreDM and T2DM. This study may identify groups at higher risk, the consequence of which is that health care providers could use this information to deliver lifestyle modification programs or pharmacological interventions within diabetes care that target the most vulnerable groups.

## Methods

### Design and sample selection

A retrospective case-control study design was used. Electronic medical records were obtained from all patients admitted to endocrinology units in four hospitals, one in each province (Jiangsu, Henan, Shanxi and Sichuan) from April 2016 to November 2016. The majority of the inpatients in the study were admitted for blood glucose adjustment or for annual chronic complication screening not for illness. This practice is very different from th at in western countries because insurance provides more generous reimbursement for inpatient care in China, which incentivizes patients to get hospitalized for even minor health conditions. Hospital discharge data obtained from electronic medical records included demographics, medical history (diagnoses), diabetes status, laboratory tests, drug treatments and blood glucose records. The Scientific Research Committees and Ethics Committees of the four hospitals approved the study.

There were 217 patients with PreDM in the data base. Age and sex -matched T2DM patient records (*n* = 900) were obtained from the database of the four centers for the calculation of 10-year ICVD risk. We included in the analysis those who were ≥ 35 years of age and had a diagnosis of PreDM or T2DM confirmed using a screening 2-h oral glucose tolerance test (OGTT). Participants were diagnosed with PreDM according to American Diabetes Association criteria: impaired fasting glucose (5.6 to 6.9 mmol/L [100 to 125 mg/dL]) and/or impaired oral glucose tolerance (7.8 to 11.1 mmol/L [140 to 199 mg/dL]. Participants were diagnosed with T2DM according to Guidelines for the Prevention and Treatment of T2DM in China (2013 Edition) [16], with typical symptoms of diabetes, random plasma glucose ≥11.1 mmol/L, or fasting plasma glucose (FPG) ≥ 7.0 mmol/L, or 2-h plasma glucose level of ≥200 mg/dL (11.1 mmol/L) during a 75-g OGTT or hemoglobin A1c (HbA1c) level ≥ 6.5%. Exclusion criteria were pregnancy or lactation, liver or kidney dysfunction, malignant tumor, mental illness, or diagnosis of coronary heart disease or ischemic stroke.

### Collection of clinical and laboratory parameters

Participants underwent anthropometric measurements, questionnaires and blood drawing at baseline. Smoking was defined as daily consumption of more than one cigarette/day for > 1 year [17]. Weight (kg) and height (cm) were determined using a standard hospital balance scale and a metal ruler; participants wore light clothing and no shoes. Waist circumference was measured at the level of the umbilicus with the patient standing and breathing normally, measured twice and averaged. BMI was calculated as body weight (kg) divided by height (m^2^). Overweight was defined using the Asian standard as BMI of 24 kg/m^2^ or greater [18].

Systolic and diastolic blood pressure (SBP, DBP) were taken on the dominant arm in a sitting position using a standard manual sphygmomanometer and an adult size cuff [19]. Hypertension was defined as the absence of antihypertensive drugs and SBP ≥140 mmHg and/or DBP ≥90 mmHg on three different days or by a previous diagnosis of hypertension with current antihypertensive medication [20]. Awareness of hypertension was defined as a participant’s self-report of diagnosed high BP. Treated hypertension was defined as antihypertensive drugs being used currently, and controlled hypertension was defined as a participant’s report of antihypertensive treatment together with a clinical measurement of SBP < 140 mmHg and DBP < 90 mmHg [21].

Total cholesterol (TC), triglycerides (TG), HDL and low-density lipoprotein cholesterol (LDL) were measured after a 10-h fast, using a standardized and reliable method, an automatic biochemical analyzer. Hyperlipidemia was defined as TC > 5.18 mmol/L or/and TG > 1.70 mmol/L. [22] Awareness of hyperlipidemia was defined as a self-report of any prior diagnosis of hyperlipidemia by a medical doctor. Treatment of hyperlipidemia was defined as use of pharmacological treatment to manage hyperlipidemia. Participants were considered to have controlled hyperlipidemia if the TC was < 5.18 mmol/L and TG was < 1.70 mmol/L after treatment.

FPG and 2-h plasma glucose (2hPG) concentrations were measured by glucose oxidase methods. HbA1c was measured using the high performance liquid chromatography method, and fasting serum insulin (FSI) concentration was measured using an electrochemiluminescence assay. Homeostatic model assessment of β-cell function (HOMA-β) was calculated as HOMA-β = 20 × FSI/ (FPG-3.5). Homeostatic Model Assessment of Insulin Resistance was calculated as HOMA-IR = (FPG × FSI)/22.5 [23].

### Calculation of absolute ICVD risk engine based on clinical and biochemical characteristics

The 10-year ICVD risk scores of participants were evaluated using the ICVD risk engine [12]. The items included: records of sex (male or female), age (≥35 years), BMI, current smoking status (no or yes), SBP, TC and diabetes [16]. A risk value was calculated using these seven indices to get an absolute 10-year ICVD risk (Appendix). According to the risk value, participants were divided into extremely high risk (≥40%), high risk (20–40%), moderate risk (10–20%), low risk (5–10%) and extremely low risk (< 5%) [24]. Therefore, the commonly used cut-off value of > 10% indicates moderate or greater risk [24].

### Statistical analysis

Epidata 3.1 and SPSS22.0 were used. Continuous variables are presented as the mean ± SD, whereas categorical variables are presented as frequencies and percentages. Data were tested for normality of distribution by the Kolmogorow–Smirnow test. If data were not normally distributed, the respective non-parametric test was used. Sex differences between baseline characteristics of those with PreDM and T2DM were analyzed using t-tests for independent groups or a non-parametric test and chi-square for categorical variables (smoking and > 10% ICVD risk). We calculated the 10-year ICVD risk of each individual using the risk engine technique. The differences in 10-year absolute ICVD risk according to PreDM, T2DM < 1 year, T2DM 1–5 years or T2DM ≥5 years and sex were analyzed using ANOVA. Differences in prevalence, awareness, treatment and control for hypertension and hyperlipidemia in males or females with T2DM were analyzed using chi-square. A *p* value of ≤.05 was considered statistically significant.

## Results

### Sample characteristics

Baseline clinical and biochemical characteristics are presented (Table 1). PreDM patients included 77 males and 140 females and those with T2DM included 300 males and 600 females. When compared to PreDM females, males with PreDM had significantly higher 10-year ICVD risk. In contrast, the opposite pattern of 10-year ICVD risk was observed in T2DM; males had significantly lower 10-year ICVD risk. Also, compared to T2DM females, males with T2DM had a lower proportion with moderate or greater ICVD risk (p<0.001).

**Table 1:** Baseline clinical and biochemical characteristics among Chinese patients with PreDM or T2DM

Variable	PreDM			T2DM		*p* value
	Male (*n* = 77)	Female (*n* = 140)	p value	Male (*n* = 300)	Female (*n* = 600)	
Age (years)	61.55 ± 5.18	61.36 ± 5.36	0.127	61.13 ± 4.46	61.51 ± 6.53	0.082
BMI (kg/m^2^)	24.89 ± 2.67	24.85 ± 3.20	0.299	24.68 ± 3.47	25.12 ± 3.76	0.148
WC (cm)	87.75 ± 8.44	85.07 ± 9.02	0.015	89.49 ± 9.05	84.92 ± 8.67	<0.001
Smoking (%)	31(40.26)	1(0.71)	<0.001	128(42.67%)	11(1.83%)	< 0.001
SBP (mmHg)	134.91 ± 16.82	132.48 ± 16.28	0.142	135.29 ± 20.05	135.23 ± 18.45	0.830
DBP (mmHg)	79.35 ± 11.93	79.70 ± 10.82	0.977	80.31 ± 11.09	79.09 ± 9.99	0.146
TC (mmol/L)	5.03 ± 0.89	5.24 ± 1.00	0.087	4.33 ± 1.17	4.68 ± 1.24	< 0.001
TG (mmol/L)	1.78 ± 0.94	1.85 ± 1.02	0.675	1.83 ± 1.30	1.95 ± 1.40	0.102
HDL (mmol/L)	1.40 ± 0.39	1.51 ± 0.37	0.072	1.09 ± 0.31	1.24 ± 0.54	< 0.001
LDL (mmol/L)	3.00 ± 0.78	3.10 ± 0.72	0.367	2.78 ± 0.87	2.92 ± 1.20	0.072
FPG (mmol/L)	5.89 ± 0.58	5.86 ± 0.59	0.890	8.02 ± 2.57	8.29 ± 2.92	0.305
2hPG (mmol/L)	7.96 ± 1.88	7.73 ± 1.54	0.257	16.01 ± 5.01	16.52 ± 4.98	0.216
HbA1c (%)	5.99 ± 0.36	5.94 ± 0.34	0.328	9.23 ± 2.51	9.10 ± 2.49	0.402
FSI (μIU/ml)	10.39 ± 4.58	11.46 ± 4.00	0.114	8.81 ± 6.35	10.24 ± 7.97	0.007
HOMA-β	61.13 ± 26.97	67.41 ± 23.56	0.114	51.59 ± 47.94	57.41 ± 53.22	0.124
HOMA-IR	2.70 ± 1.19	3.00 ± 1.13	0.145	3.20 ± 2.90	3.72 ± 3.27	0.005
10-year ICVD risk (%)	7.54 ± 6.71	4.97 ± 5.20	<0.001	9.64 ± 9.58	11.77 ± 10.56	0.014
Moderate or greater ICVD risk n (%)	16(20.78)	23(16.43)	0.424	71(23.67)	283(47.17)	<0.001

Values are mean ± SD. BMI, Body mass index; WC, waist circumference; SBP, Systolic blood pressure; DBP, Diastolic blood pressure; TC, Total cholesterol; TG, Triglyceride; HDL, High density lipoproteins; LDL, Low density lipoproteins FPG, Fasting plasma glucose; 2hPG, 2-h plasma glucose; HbA1c, Glycated hemoglobin; FSI, Fasting serum insulin; HOMA-β, Homeostasis model assessment of beta cell function; HOMA-IR, Homeostasis model assessment of insulin resistance

### 10-year ICVD risk in male and female patients with PreDM or T2DM

**Fig. 1:**
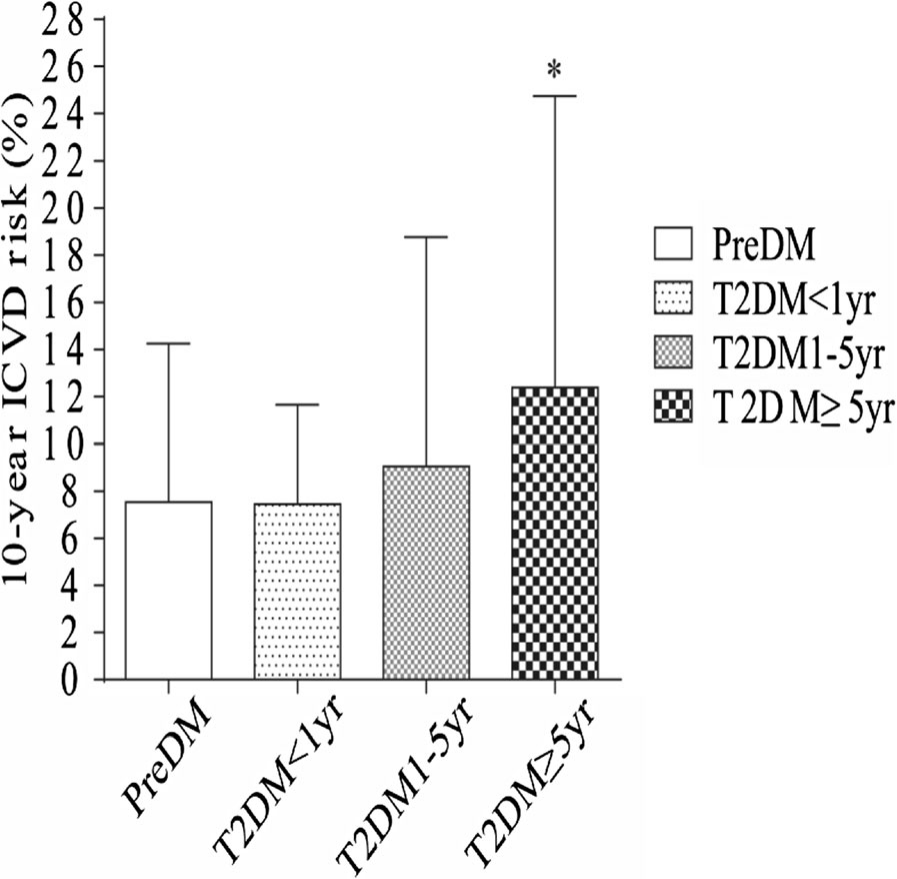
Assessment of 10-year ICVD risk in males with PreDM and T2DM.Values are mean ± SD. *p ≤ 0.05, compared T2DM to PreDM in male patients

**Fig. 2:**
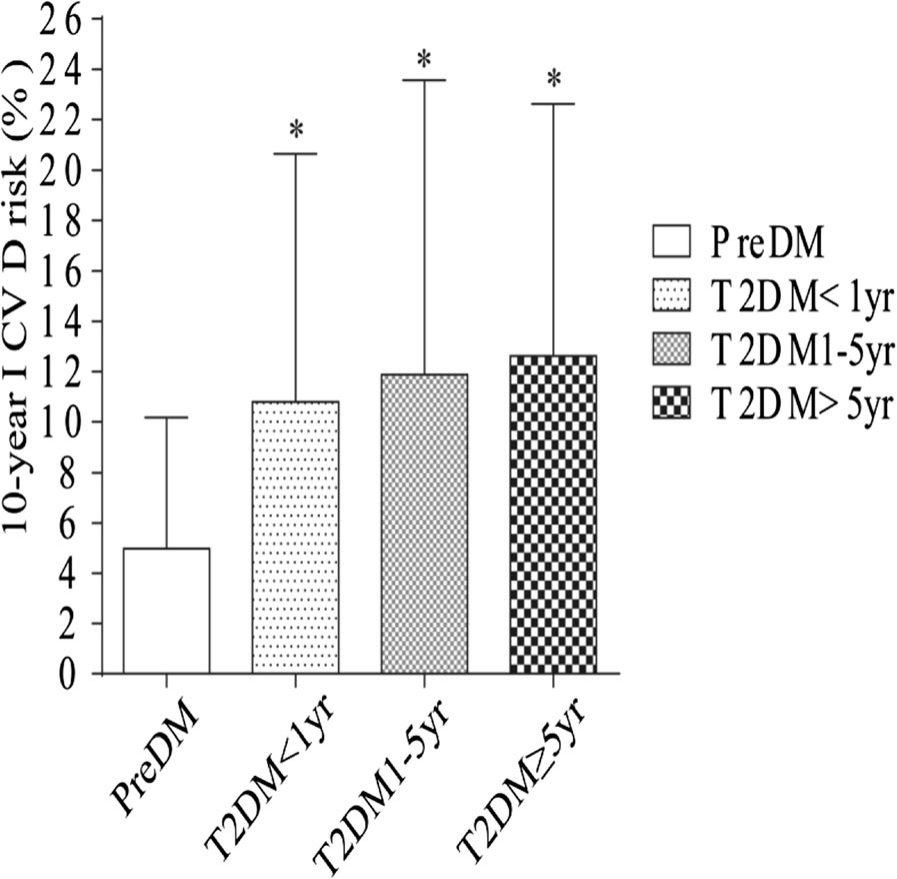
Assessment of 10-year ICVD risk in females with PreDM and T2DM. Values are mean ± SD. *p ≤ 0.05, compared T2DM to PreDM in female patients

We divided the T2DM patients into 3 subgroups according to their diabetes duration: T2DM < 1 year, T2DM 1–5 years or T2DM ≥5 years. The pattern of 10-year ICVD risk across PreDM and T2DM was dissimilar in males and females (Fig. 1 and Fig. 2). When compared to PreDM males, males with T2DM < 1 year, and with T2DM 1–5 years had no difference in 10-year ICVD risk, but had higher ICVD risk with T2DM ≥5 years (*p* < 0.05). Compared to PreDM females, females with T2DM in all subgroups had higher ICVD risk (p < 0.05). These findings indicate different cut-off points for 10-year ICVD risk in male and female patients.

### Prevalence, awareness, treatment and control of hypertension and hyperlipidemia in male and female Chinese with T2DM

The awareness, treatment and control of hypertension and hyperlipidemia overall (*n* = 900) were: 78.60% were aware of the hypertension diagnosis, 65.38% were receiving treatment and 31.10% had hypertension controlled, whereas those for hyperlipidemia were lower (29.15%, 8.30% and 3.47%, respectively). These outcomes in Chinese with T2DM are presented according to sex in Table 2. Females with T2DM had a significantly higher prevalence, awareness, treatment and control of hypertension and also prevalence, awareness and treatment of hyperlipidemia than males with T2DM . The numbers for awareness, treatment and control of hypertension and hyperlipidemia were very low in both males and females.

**Table 2:** Prevalence, awareness, treatment and control of hypertension and hyperlipidemia in T2DM patients according to sex (*n* = 900)

Risk factor	Prevalence n (%)		*p* value	Awareness n (%)		*p* value	Treatment n (%)		*p* value	Control n (%)		*p* value
	Male	Female		Male	Female		Male	Female		Male	Female	
Hypertension	197(21.89)	401(44.56)	< 0.001	163(27.26)	307(51.34)	< 0.001	132(22.07)	259(43.31)	< 0.001	65(10.87)	121(20.23)	< 0.001
Hyperlipidemia	158(17.56)	360(40.00)	< 0.001	60(11.58)	91(17.57)	0.006	11(2.12)	32(6.18)	0.001	7 (1.35)	11(2.12)	0.342

## Discussion

An interesting aspect of our study is that the 10-year ICVD risk was higher in males with PreDM, whereas there is a definite converse difference in T2DM. A similar finding [25] was observed in a previous studythat showed, at a given glycemic state, the absolute glycemic CVD risk was higher in males than in females.  However, this difference narrowed when progressing from normal glycaemia to newly diagnosed diabetes, and the relative risk of CVD was higher in females with DM in comparison with male counterparts. Additionally, males with T2DM compared to females had a significantly lower proportion with a moderate or greater risk category. This is inconsistent with a Caribbean study, which reported a relatively higher proportion of male patients at > 15% 10-year ICVD risk compared to females (60.4% vs 30.6%) [26]. This difference in results might be due to different models and algorithms for CVD risk calculation with different risk categories in different races. Our study suggested that Chinese female T2DM patients are at higher 10-year ICVD risk, and great attention should be paid to this finding.

 Compared to PreDM, we found that a significantly high 10-year ICVD risk occurred in females with T2DM at < 1 year, yet this finding was observed later in males with T2DM at > 5 years. The implication is that earlier intervention programs should be implemented for female patients with T2DM. There are two reasons that may account for the 10-year ICVD pattern being worse in females. First, recent accumulating evidence demonstrates that diabetes alters estrogen-related protective mechanisms and causes pronounced adverse changes in cardiovascular risk factors leading to enhanced atherogenesis in females [27, 28]. In our study, the average age of females with T2DM was about 61 years; gradually decreasing estrogen levels may indirectly result in higher ICVD risk. Second, in a cross-sectional analysis,females were less likely than males to meet the goals for LDL cholesterol; this suggests the need for gender-specific approaches [29]. A similar result, found in this research population, was that females with T2DM had a higher prevalence rate of hypertension and hyperlipidemia. Additionally, females with T2DM compared to males had significantly less optimal values for HOMA-IR. Insulin resistance levels are important and should be highlighted because insulin resistenace  has been shown to be an independent risk factor for cardiovascular events [30].

Hypertension and hyperlipidemia are well-established causal risk factors for ischemic cardiovascular disease [31]. In the current study, the prevalence, awareness, treatment and control of hypertension overall were 66.44%, 78.60%, 65.38% and 31.10%, respectively. More than half of the T2DM patients had hypertension. A similar result was obtained in a US study that found that the frequency of hypertension was 63% in 2012 among both prevalent and newly-diagnosed T2DM cohorts [32]. Although the awareness and treatment rate of hypertension in our study signals a need for more interventions in both males and females, it is on the control rate that researchers and clinicians in China must first focus. In addition, it should be noted that encouraging primary care physicians and other public healthcare professionals to continue to expand their efforts to control high blood pressure is necessary for Chinese patients. We also observed that in the current study, hyperlipidemia rates of prevalence, awareness, treatment and control were 57.56%, 29.15%, 8.30% and 3.47%, respectively. A similar result on prevalence was found in a US study; it reported that the frequency of hyperlipidemia with T2DM was 56.9% [32].The awareness, treatment and control of hyperlipidemia were not at high levels, possibly suggesting that people do not pay attention to their health or people with hyperlipidemia do not adhere to prescribed treatments, even after becoming aware of hyperlipidemia. Therefore, **i**t is apparent that a national hyperlipidemia education program to promote community- and clinic-based serum lipid screening is urgently needed in China.

The strength of the current study is that this was the first study to report the absolute 10-year ICVD risk assessment in Chinese patients with PreDM or T2DM. The current study has several limitations. First, the current study lacked data on the awareness, treatment and control of hypertension and hyperlipidemia in those with PreDM, and it also lacked data on physical activity, sedentary behavior time and specific medical treatments, which are strongly associated with the development of the metabolic syndrome, T2DM and CVD. Second, data on smoking, hypertension and hyperlipidemia were self-reported.

## Conclusions

In conclusion, the current study demonstrated that different cut-off points exist for focusing on ischemic cardiovascular diseases: before 5 years for males and less than 1 year for females with T2DM. There is a greater need for cardiovascular risk reduction programs for females with T2DM, as soon as they are diagnosed. Also, given the higher prevalence of hypertension and hyperlipidemia in females, significant resources focused on them must be expended. Diabetes education in males is also needed before 5 years to be able to improve awareness, treatment and control of hypertension and hyperlipidemia, in particular. A key to reducing 10-year risk is continued intensive follow-up, which may or may not be possible in China with so many Chinese affected.

## Figures and Tables

**Table:**
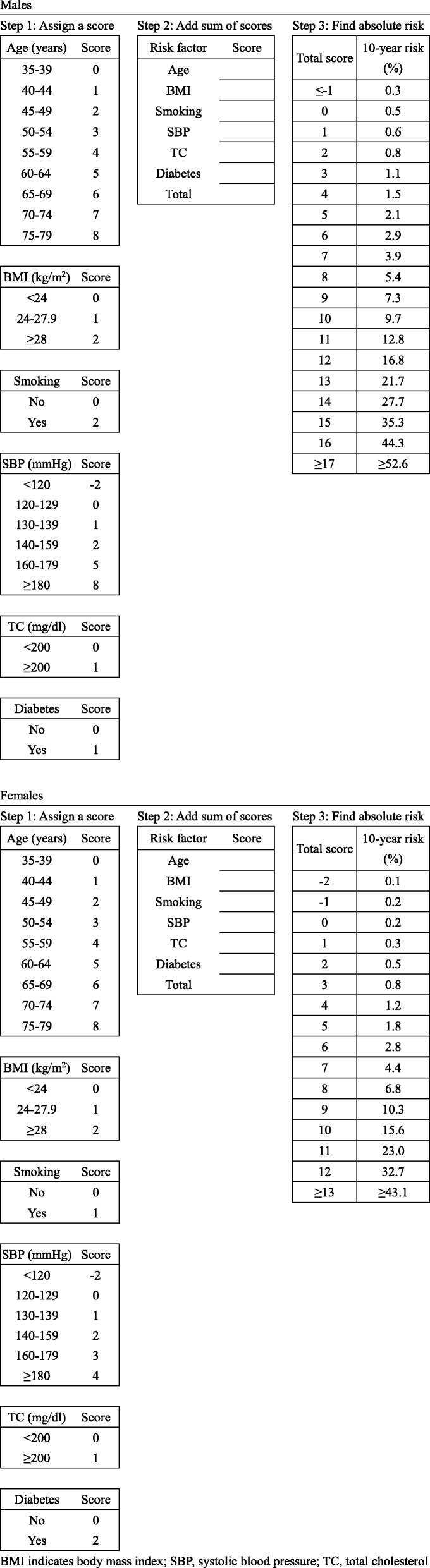
Evaluation sheet for estimating 10-year risk of ischemic cardiovascular disease incidence for males and females

## Data Availability

The datasets used and/or analysed during the current study are available from the corresponding author on reasonable request.

## Abbreviations

2hPG: 2-h plasma glucose
BMI: Body mass index
DBP: Diastolic blood pressure
FPG: Fasting plasma glucose
FSI: Fasting serum insulin
HbA1c: Glycated hemoglobin
HDL: High density lipoproteins
HOMA-IR: Homeostasis model assessment of insulin resistance
HOMA-β: Homeostasis model assessment of beta cell function
LDL: Low density lipoproteins
SBP: Systolic blood pressure
TC: Total cholesterol
TG: Triglyceride
WC: Waist circumference
