# Correction to: Sex differences in 10-year ischemic cardiovascular disease risk prediction in Chinese patients with prediabetes and type 2 diabetes

**DOI:** 10.1186/s12872-020-01470-9

**Published:** 2020-04-16

**Authors:** Lihong Yang, Anne F. Fish, Yuanyuan Zhu, Xiaodan Yuan, Jianing Li, Xiaoyun Wang, Li Yuan, Zhumin Jia, Chao Liu, Chenyun Xu, Qingqing Lou

**Affiliations:** 1 Department of Health Education, Affiliated Hospital of Integrated Traditional Chinese and Western Medicine, Nanjing University of Chinese Medicine, #100 Hongshan Road, Qi xia District, Nanjing, 210028 Jiangsu Province China; 2 Jiangsu Province Academy of Traditional Chinese Medicine, 100 Shizi Street, Hongshan Rd, Qi Xia District, Nanjing, Jiangsu Province China; 3 Eastern Branch of Suzhou Municipal Hospital, 16 Baita West Rd, Gu Su District, Suzhou, Jiangsu Province China; 4 College of Nursing (ISP Program), University of Missouri-St. Louis, One University Blvd, St. Louis, MO M/C 529 USA; 5 Nursing College, Nanjing University of Chinese Medicine, 138 Xianlin Rd, Qi Xia District, Nanjing, Jiangsu Province China; 6 Department of Endocrinology, Shanxi Provincial People’s Hospital, 29 Twin Towers Temple Street, Taiyuan, Shanxi Province China; 7 Department of Endocrinology, West China Hospital, West China School of Medicine, Sichuan University, 37 Guoxue Lane, Wu Hou District, Chengdu, Sichuan Province China; 8 Department of Endocrinology, Affiliated Hospital of Henan University of Science and Technology, 24 Jinghua Rd, Luoyang, Henan Province China; 9 Hainan General Hospital-Longhua, #8 Longhua Rd, Longhua District Hainan, Haikou, 570102 China

**Correction to: BMC Cardiovasc Disord (2019) 19:301.**

**https://doi.org/10.1186/s12872-019-1232-y**

After the publication of the original article [1], we were notified that one of the corresponding author’s name and her related institution were wrongly spelled.

Yunchen Xu should be replaced with author’s Given name Chenyun, and Family name Xu.

Her institution “Hainan People’s Hospital-Longhua, #8 Longhua Rd, Longhua District Hainan, Haikou 570102, China” should be instead indicated as “Hainan General Hospital-Longhua, #8 Longhua Rd, Longhua District Hainan, Haikou 570102, China”.
